# Associations of primiparous pre-pregnancy body mass index and gestational weight gain with cesarean delivery after induction: a prospective cohort study

**DOI:** 10.3389/fmed.2024.1453620

**Published:** 2024-08-30

**Authors:** Shi Lin, Chunzhi Xie, Anyi Teng, Xiaotian Chen, Yan Li, Yangyang Zhang, Hui Zhang, Ting Sun

**Affiliations:** ^1^Department of Gynaecology and Obstetrics, Maternal and Child Health Hospital, Songjiang, Shanghai, China; ^2^Department of Clinical Epidemiology, Children’s Hospital of Fudan University, Shanghai, China

**Keywords:** cesarean delivery after induction, induction of labor, body mass index, gestational weight gain, primiparous woman

## Abstract

**Objective:**

The effects of Pre-pregnancy body mass index (BMI) and gestational weight gain (GWG) in primiparas remain unclear. This study examines the associations of pre-pregnancy BMI and GWG with cesarean delivery after induction (CDaI) in primiparous women.

**Methods:**

This prospective cohort study included 3,054 primiparous women. We recorded pre-pregnancy BMI, first, second, and third trimester weight values, as well as instances of CDaI and other pregnancy outcomes. We analyzed the associations of pre-pregnancy BMI and GWG with CDaI by conducting a multivariate logistic regression analysis after adjusting for covariates, and adjusted risk ratios (aRR) and 95% confidence intervals were reported.

**Results:**

We recorded 969 CDaIs. In the vaginal delivery group, each increase of 1 standard deviation in the pre-pregnancy BMI was correlated with a 6% increase in the CDaI risk [aRR (95% CI), 1.06 (1.01–1.11)]. Each increase of 1 standard deviation in the rate of weight gain during the entire pregnancy was correlated with a 21% increase in the CDaI risk [aRR (95% CI), 1.21 (1.14–1.29)]. Compared to women with a normal weekly GWG in the second and third trimester, those with slow GWG had a 19% increased risk of CDaI [aRR (95% CI), 1.19 (1.01–1.37)]. The subgroup analysis results showed that increases in pre-pregnancy BMI could increase the CDaI risk regardless of the induction method.

**Conclusion:**

High pre-pregnancy BMI, excessive GWG, and rapid first trimester weight gain are risk factors for CDaI in primiparous women. Excessive first trimester weight gain, may associated with increased risks of CDaI in primiparous women.

## 1 Introduction

Cesarean delivery after induction (CDaI) is used to describe instances in which a decision is made to switch from vaginal delivery after induction to cesarean section due to fetal distress, abnormal labor circumstances, or other clinical indications. Although induction of labor (IoL) itself does not increase the rate of cesarean sections (1), the CDaI incidence has increased alongside increases in IoL rates (2, 3) and has reached a percentage as high as 29.4% (4). CDaI is an important approach for resolving difficult labor situations, but the procedure carries greater risks than a planned cesarean delivery (PCD). The short-term risks of CDaI may include maternal uterine incision tears, postpartum hemorrhage, puerperal infection, the need for blood transfusion, hysterectomy, venous thromboembolism, neonatal complications, and infant sepsis (5, 6). In the long term, CDaI may lead to pregnancies at the uterine scar site, placenta previa, and the need for repeat cesarean sections as a form of delivery. The medical cost of CDaI is significantly higher than that of natural delivery or PCD, and a CDaI may have potential physiological and negative mental effects on the pregnant woman and her family members (7). Decreasing the occurrence of CDaI cannot be achieved solely by preventing IoL; instead identifying pre-pregnancy and prenatal risk factors for interventions is necessary.

Risk factors associated with CDaI have been reported, including older maternal age, shorter height, pre-pregnancy obesity, greater gestational weight gain (GWG), cervical length, neonatal gender, and weight (8, 9). Pre-pregnancy obesity and weight gain during pregnancy are important CDaI modifiable risk factors (4, 9). However, the publications on relevant population studies have limitations. For one, most studies are restricted to primiparas (10). Moreover, the published reports have focused on a single IoL method (11). There is a lack of research on the associations of CDaI with weight gain and weight gain rate in primiparas before pregnancy or at different stages during pregnancy. We designed a prospective cohort study to evaluate the associations between CDaI in primiparas and their pre-pregnancy BMI, GWG, and weight gain rates in different pregnancy periods. These findings may provide some novel evidence to implement strategies for the eventual reduction of CDaI among primiparas.

## 2 Materials and methods

### 2.1 Participants

The participants of this study were pregnant women receiving prenatal check-ups and delivered at the Songjiang District Maternal and Child Health Hospital in Shanghai from January 2020 to December 2022. Primiparas with medical record data on maternal pre-pregnancy BMI, GWG, IoL, and way of delivery were included. Those who met any one of the following criteria were excluded: multiparous women; gestational age < 37 weeks; multiple gestation; hyperemesis gravidarum causing weight loss in early pregnancy; non-cephalic presentation; premature rupture of membranes; cesarean section with abnormal birth canal, scarred uterus, relative or absolute indications for cesarean section such as placenta previa, lack of desire for attempting vaginal delivery; disagreement with induction of labor; or allergies to labor induction drugs. The research protocol was approved by the hospital ethics committee (approval number: 20210201), and all participants signed written informed consent forms.

### 2.2 Definition of outcomes

We defined CDaI as a case in which a pregnant woman undergoing IoL develops indications for cesarean delivery (CD), such as abnormal fetal heart rate, abnormal labor, abnormal fetal position, or chorioamnionitis. CD were performed after obtaining the consent of the pregnant woman and her family. IoL refers to the use of drugs and other means to initiate labor for the purpose of delivery before the onset of natural labor. This can include administration of intravenous oxytocin, artificial rupture of membranes, insertion of cervical water sacs, administration of prostaglandin preparations, or other interventions. The indications and timing of IoL in this study were based on the 2020 Chinese “Expert Consensus on Timing of Termination of Pregnancy Complications and Comorbidities” (12).

### 2.3 Exposures and covariates

The main exposures in this study were pre-pregnancy BMI and GWG, and the GWG rate. Pre-pregnancy BMI was provided by participants and verified against weight data during pre-pregnancy examinations. We classified the GWG of participants as either inadequate, within guidelines, or excessive according to the Chinese Nutrition Society standards “Chinese women gestational weight gain monitoring and evaluation” (T/CNSS-009-2021, Supplementary Table S1) (13). The height and weight of pregnant women were measured using hospital equipment (Omron weighing scale HNH-219). During measurements, the participants took their shoes and thick coats off, and the height and weight values were precise to 1 decimal place.

We included covariates such as age, height, gestational age, IoL method, labor analgesia (binary variable, yes or no), Doula delivery (binary variable, yes or no), gestational diabetes mellitus (GDM, binary variable, yes or no), and hypertensive disorders of pregnancy (HDP, binary variable, yes or no) for this study, which were leveraged from the electronic medical record system. Doulas provide professional and humane services to mothers by full-time dedicated personnel to achieve sustained and significant non-drug labor analgesia. All participants underwent screening for gestational diabetes mellitus (GDM), with a 75-g oral glucose tolerance test (OGTT) performed at 24 to 28 weeks of gestation. The blood glucose thresholds during fasting, and at 1 and 2 h after oral glucose were 5.1, 10.0, and 8.5 mmol/L, respectively. GDM was diagnosed if the blood glucose values reached or exceeded these standards at any time point. We recorded instances of hypertensive disorders of pregnancy (HDP), including gestational hypertension, preeclampsia, chronic hypertension, and chronic hypertension with superimposed preeclampsia.

### 2.4 Statistical analysis

We expressed normally distributed continuous variables as means (standard deviations [SDs]) and non-normally distributed variables as medians (inter-quartile ranges [IQRs]). Normality was assessed by visual inspection of frequency histograms. We summarized categorical variables as frequencies and percentages, and compared using the chi-square tests. Comparisons between data for continuous variables in the vaginal delivery group and the CDaI group were performed using 2-tailed unpaired Student’s *t*-tests or Mann–Whitney U tests depending on normality. Missing data for exposures and covariates were imputed by multiple imputations using chained equations based on the assumption that the data were missing at random. Ten imputed data sets were generated, with the results combined using the Rubin rule (14).

Our primary aim was to investigate the associations of GWG levels with the CDaI risk. We used generalized linear models with a binomial family with a log link function, treating GWG (rescaled by 1 SD) as continuous variables to estimate crude and adjusted risk ratios (aRRs) and 95% confidence intervals (CIs). The adjusted covariates included maternal age, gestational week, assisted reproductive technology, epidural labor analgesia, doula-accompanied delivery, GDM, and HDP. In secondary analyses, we investigated the associations of Pre-pregnancy BMI, GWG, and GWG rate at the first trimester and during the total with the CDaI risk. Considering the IoL method was one of the most common clinical factors related to CDaI, we conducted sub-group analyses in the pregnant women undergoing different IoL methods, according to the 2014 Chinese “Guidelines for Cervical Ripening and Induction of Labor in Late Pregnancy” (15). Detailed definitions of the IoL method were summarized in Supplementary Table S2. As the sample size in the IoL method 3 sub-group was small, we performed sub-group analyses only IoL method 1 and IoL method 2. In addition, to provide more robust findings, we performed subgroup analysis stratified by BMI category in evaluation of the associations of GWG with CDaI risk. Statistical analyses were performed by Stata version 16.0, and all statistical tests were 2-sided at a significance level of 0.05.

## 3 Results

A total of 3,467 participants were recruited for this study. After excluding women who underwent PCD without IoL (*n* = 307), those who delivered prematurely (*n* = 84), or who had stillborns (*n* = 4), participants who did not undergo prenatal examination (*n* = 18), 3,054 pregnant women were finally included in the analysis. CDaI was performed in 969 participants (31.7%), and the remaining participants underwent vaginal delivery.

The mean (SD) age of participants in this study was 28.1 years (±3.6) while the mean (SD) ages of women in the vaginal delivery and CDaI groups were 27.8 years (3.5) and 28.4 years (±3.7), respectively. The mean age of the vaginal delivery group was lower than that in the CDaI group (*p* < 0.001) (Table 1). The mean gestational age for participants in the vaginal delivery group who received IoL [39.6 weeks (±1.0)] was similar to that of the CDaI group [39.7 weeks (±1.0)]. Compared with the vaginal delivery group, the proportions of participants who underwent assisted reproductive technology, had a gestational hypertensive disease, underwent Foley bulb induction, and underwent artificial membrane rupture were all significantly higher in the CDaI group (3.0% vs. 5.8%, 5.5% vs. 7.5%, 14.6% vs. 25.2%, and 99.2% vs 97.1%, respectively; *p* < 0.05 for all). By contrast, the proportion of patients who underwent neuraxial labor analgesia and doula delivery was lower (82.7% vs. 59.4% and 57.1% vs. 12.1%, respectively; *p* < 0.05 for all). We found similar incidences of GDM in the two groups. The pre-pregnancy BMI, early trimester weight gain, and GWG rate of the participants in the CDaI group were significantly higher than those in the vaginal delivery group (mean [SD] 22.3 kg/m^2^ [±3.3] vs. 21.3 kg/m^2^ [±3.0], 1.7 kg [±3.6] vs. 1.4[±4.1] kg, 0.13 kg/week [±0.2] vs. 0.11[±0.3] kg/week, respectively; *p* < 0.05).

**Table 1:** Baseline characteristics of the study population.

Characteristics	Total	Vaginal delivery group	CDaI group	*P*
N	3,054	2,085	969	
Age (years), mean (SD)	28.1 (3.6)	27.8 (3.5)	28.4 (3.7)	<0.001
Missing, *n* (%)	6 (0.2)	6 (0.3)	0	
Gestation week of delivery, mean (SD)	39.6 (0.9)	39.6 (1.0)	39.7 (1.0)	0.07
Pre-pregnancy BMI (kg/m^2^), mean (SD)	21.6 (3.1)	21.3 (3.0)	22.3 (3.3)	<0.001
Underweight (BMI < 18.5 kg/m^2^), *n* (%)	383 (12.5)	298 (14.3)	85 (8.8)	<0.001
Normal weight (18.5 kg/m^2^ ≤ BMI < 24 kg/m^2^), *n* (%)	2084 (68.2)	1,437 (68.9)	647 (66.8)	
Overweight (24 kg/m^2^ ≤ BMI. < 28 kg/m^2^), *n* (%)	443 (14.5)	273 (13.1)	170 (17.5)	
Obese (BMI ≥ 28 kg/m^2^), *n* (%)	144 (4.7)	77 (3.7)	67 (6.9)	
ART, *n* (%)	119 (3.9)	63 (3.0)	56 (5.8)	<0.001
Missing, *n* (%)	8 (0.3)	2 (0.1)	6 (0.6)	
Epidural labor analgesia, *n* (%)	2,300 (75.3)	1724 (82.7)	576 (59.4)	<0.001
Missing, *n* (%)	20 (0.7)	3 (0.1)	17 (1.8)	
Doula-accompanied delivery, *n* (%)	1,307 (42.8)	1,190 (57.1)	117 (12.1)	<0.001
Missing, *n* (%)	11 (0.4)	1 (0.05)	10 (1.4)	
GDM, *n* (%)	671 (22.0)	446 (21.4)	225 (23.2)	0.26
HDP, *n* (%)	188 (6.2)	115 (5.5)	73 (7.5)	0.031
Foley bulb, *n* (%)	549 (18.0)	305 (14.6)	244 (25.2)	<0.001
Missing, *n* (%)	12 (0.4)	2 (0.1)	10 (1.4)	
Amniotomy, *n* (%)	3,009 (98.5)	2068 (99.2)	941 (97.1)	0.013
Missing, *n* (%)	9 (0.3)	3 (0.1)	6 (0.9)	
Oxytocin, *n* (%)	2,641 (86.5)	1822 (87.4)	819 (84.5)	0.14
Missing, *n* (%)	10 (0.3)	2 (0.1)	8 (1.1)	
GWG				
At first trimester (kg), mean (SD)	1.5 (3.9)	1.4 (4.1)	1.7 (3.6)	0.042
Inadequate, *n* (%)	794 (26.0)	574 (27.5)	220 (22.7)	0.007
Within guidelines, *n* (%)	1,175 (38.5)	801 (38.4)	374 (38.6)	
Excessive, *n* (%)	1,085 (35.5)	710 (34.1)	375 (38.7)	
Total pregnancy (kg), mean (SD)	11.4 (7.0)	11.3 (7.1)	11.7 (6.8)	0.10
Inadequate, *n* (%)	885 (29.0)	626 (30.0)	261 (26.9)	0.14
Within guidelines, *n* (%)	1,068 (35.0)	578 (27.7)	259 (26.7)	
Excessive*, n* (%)	1,101 (36.1)	881 (42.3)	449 (46.3)	
Rate of GWG				
At first trimester (kg/week), mean (SD)	0.11 (0.3)	0.11 (0.3)	0.13 (0.2)	0.044
At second and third trimester (kg/week), mean (SD)	0.37 (0.3)	0.37 (0.3)	0.38 (0.2)	0.67
Low, *n* (%)	922 (30.2)	641 (30.7)	281 (29.0)	0.55
Normal, *n* (%)	846 (27.7)	578 (27.7)	268 (27.7)	
High, *n* (%)	1,286 (42.1)	866 (41.5)	420 (43.3)	

BMI, body mass index; GWG, gestational weight gain; HDP, hypertensive disorders of pregnancy; ART, assisted reproductive techniques; SD, standard deviation.

As shown in Table 2, each 1 kg/m^2^ increase in the pre-pregnancy BMI was associated with a 6% increase in the CDaI risk, with a crude RR (95% CI) of 1.06 (1.04–1.08), *p* < 0.001. After adjusting for covariates, the results remained significant with an aRR (95% CI) of 1.05 (1.03–1.07), *p* < 0.001. Compared to participants with normal pre-pregnancy weight, obese participants had a 35% increased risk of CDaI with an aRR (95% CI) of 1.35 (1.03–1.76), and *p* = 0.027. Conversely, the risk of CDaI in underweight participants shown a 30% decrease with an aRR (95% CI) of 0.70 (0.56–0.89), and *p* = 0.003.

**Table 2:** Association analyses of pre-pregnancy BMI and GWG with CDaI risk.

Variables	Case/total (%)	Crude *RR*	*P*	Adjusted *RR*	*P*
Pre-pregnancy BMI		1.06 (1.04–1.08)	<0.001	1.05 (1.03–1.07)	<0.001 ^a^
Underweight (BMI < 18.5 kg/m^2^), *n* (%)	85/383 (22.1)	0.71 (0.57–0.89)	0.004	0.70 (0.56–0.89)	0.003 ^a^
Normal weight (18.5 kg/m^2^ ≤ BMI < 24 kg/m^2^), *n* (%)	647/2084 (31.0)	Ref		Ref	
Overweight (24 kg/m^2^ ≤ BMI < 28 kg/m^2^), *n* (%)	170/443 (38.4)	1.24 (1.04–1.46)	0.014	1.13 (0.95–1.35)	0.15 ^a^
Obese (BMI ≥ 28 kg/m^2^), *n* (%)	67/144 (46.5)	1.50 (1.16–1.93)	0.002	1.35 (1.03–1.76)	0.027 ^a^
GWG					
At first trimester (per 1 SD, kg)		1.05 (0.99–1.10)	0.09	1.06 (1.01–1.11)	0.019 ^b^
Inadequate, *n* (%)	220/794 (27.7)	0.87 (0.74–0.98)	0.031	1.03 (0.87–1.22)	0.72 ^b^
Within guidelines, n (%)	374/1175 (31.8)	Ref		Ref	
Excessive, *n* (%)	375/1085 (34.6)	1.08 (0.94–1.25)	0.26	1.25 (1.08–1.44)	0.003 ^b^
Total pregnancy (per 1 SD, kg)		1.17 (1.10–1.24)	<0.001	1.21 (1.14–1.29)	<0.001 ^b^
Inadequate, *n* (%)	259/885 (29.3)	0.87 (0.74–1.03)	0.10	1.11 (0.95–1.31)	0.19 ^b^
Within guidelines, *n* (%)	357/1068 (33.4)	Ref		Ref	
Excessive, *n* (%)	353/1101 (32.1)	0.96 (0.83–1.11)	0.58	1.07 (0.92–1.24)	0.37 ^b^
Rate of GWG					
At first trimester (per 1 SD, kg/week)		1.17 (0.97–1.40)	0.09	1.20 (1.03–1.39)	0.019 ^b^
At second and third trimester (per 1 SD, kg/week)		1.04 (0.82–1.32)	0.73	0.88 (0.69–1.12)	0.29 ^b^
Low, *n* (%)	281/922 (30.5)	0.96 (0.81–1.13)	0.65	1.19 (1.01–1.37)	0.040 ^b^
Normal, *n* (%)	268/846 (31.7)	Ref		Ref	
High, *n* (%)	420/1286 (32.7)	1.03 (0.88–1.20)	0.70	1.04 (0.99–1.39)	0.06 ^b^

a, adjusted for age, gestational week, ART, epidural labor analgesia, doula-accompanied delivery, GDM, HDP, Water sac, amniotomy and oxytocin; b, adjusted for pre-pregnancy BMI, age, gestational week, ART, epidural labor analgesia, doula-accompanied delivery, GDM, HDP, Foley bulb, amniotomy and oxytocin; Ref, reference group.

Each 1 SD increase in GWG during the first trimester was associated with a 6% higher risk of CDaI with an aRR (95% CI) of 1.06 (1.01–1.11), and *p* = 0.019. Compared with participants with a normal GWG in the first trimester, the risk of CDaI in participants with rapid GWG was increased by 25% with an aRR (95% CI) of 1.25 (1.08–1.44), and *p* = 0.019. The rate of GWG in the first trimester was also positively associated CDaI risk (aRR [95% CI] per 1 SD increase: 1.20 (1.03–1.39), *p* = 0.019). Each increase in weight of 1 SD throughout pregnancy was associated with a 21% increase in the risk of CDaI with an aRR (95% CI) of 1.21 (1.14–1.29), and *p* < 0.001. Compared to women with normal weekly weight gains throughout their pregnancy, those with slow weight gain during their pregnancy had a 19% increased risk of CDaI, with an aRR (95% CI) of 1.19 (1.01–1.37), and *p* = 0.040.

In the normal pre-pregnancy BMI sub-group, each 1 SD increase in GWG and excessive GWG status during the first trimester were associated with a 10 and 22% higher risk of CDaI with aRRs (95% CIs) of 1.10 (1.01–1.20) and 1.22(1.02–1.45), and *p* values of 0.027 and 0.025, respectively (Table 3). Similar positive associations of each 1 SD increase in total GWG levels and rate of GWG at the first trimester with CDaI risk were observed (aRRs [95% CIs] were 1.20[1.11–1.30] and 1.38[1.04–1.84], *p* values were < 0.001 and 0.027, respectively). In the low and high pre-pregnancy BMI sub-groups, each 1 SD increase in total GWG was associated with a 36 and 19% higher risk of CDaI with aRRs (95% CIs) of 1.36 (1.07–1.73) and 1.19 (1.05–1.34), and *p* values were 0.013 and 0.005, respectively.

**Table 3:** Association analyses for GWG with CDaI risk stratified by pre-pregnancy BMI status.

Subgroup	GWG	Cases/total (%)	Adjusted RR	*P*
Normal pre-pregnancy BMI	At first trimester (per 1 SD, kg)		1.10 (1.01–1.20)	0.027
	Inadequate, *n* (%)	259/824 (40.0)	0.99 (0.80–1.45)	0.94
	Within guidelines, n (%)	139/531 (21.5)	Ref	
	Excessive, *n* (%)	249/729 (38.5)	1.22 (1.02–1.45)	0.025
	Total pregnancy (per 1 SD, kg)		1.20 (1.11–1.30)	<0.001
	Inadequate, *n* (%)	181/603 (28.0)	1.31 (1.06–1.63)	0.013
	Within guidelines, *n* (%)	170/571 (26.3)	Ref	
	Excessive, *n* (%)	296/910 (45.7)	1.17 (0.97–1.42)	0.09
	Rate of GWG			
	At first trimester (per 1 SD, kg/week)		1.38 (1.04–1.84)	0.027
	At second and third trimester (per 1 SD, kg/week)		0.79 (0.57–1.09)	0.16
	Low, *n* (%)	198/606 (30.6)	1.23 (1.01–1.51)	0.043
	Normal, *n* (%)	183/608 (28.3)	Ref	
	High, *n* (%)	266/870 (41.1)	1.07 (0.88–1.29)	0.49
Low pre-pregnancy BMI	At first trimester (per 1 SD, kg)		1.13 (0.91–1.40)	0.24
	Inadequate, *n* (%)	31/146 (21.2)	1.10 (0.53–2.30)	0.80
	Within guidelines, n (%)	10/66 (15.1)	Ref	
	Excessive, *n* (%)	44/171 (35.7)	1.47 (0.91–2.38)	0.12
	Total pregnancy (per 1 SD, kg)		1.36 (1.07–1.73)	0.013
	Inadequate, *n* (%)	36/136 (42.3)	0.86 (0.49–1.52)	0.61
	Within guidelines, *n* (%)	21/121 (24.7)	Ref	
	Excessive, *n* (%)	28/126 (33.0)	0.95 (0.57–1.60)	0.85
	Rate of GWG			
	At first trimester (per 1 SD, kg/week)		1.52 (0.75–3.03)	0.24
	At second and third trimester (per 1 SD, kg/week)		0.80 (0.32–2.00)	0.64
	Low, *n* (%)	35/148 (41.2)	1.10 (0.65–1.88)	0.72
	Normal, *n* (%)	26/133 (30.6)	Ref	
	High, *n* (%)	24/102 (28.2)	1.09 (0.63–1.86)	0.76
High pre-pregnancy BMI	At first trimester (per 1 SD, kg)		1.03 (0.96–1.11)	0.37
	Inadequate, *n* (%)	84/205 (35.4)	1.09 (0.78–1.51)	0.62
	Within guidelines, n (%)	71/197 (30.0)	Ref	
	Excessive, *n* (%)	82/185 (34.6)	1.33 (0.97–1.82)	0.07
	Total pregnancy (per 1 SD, kg)		1.19 (1.05–1.34)	0.005
	Inadequate, *n* (%)	140/329 (59.1)	0.99 (0.72–1.35)	0.95
	Within guidelines, *n* (%)	68/193 (28.7)	Ref	
	Excessive, *n* (%)	29/65 (12.2)	0.93 (0.57–1.52)	0.77
	Rate of GWG			
	At first trimester (per 1 SD, kg/week)		1.11 (0.88–1.41)	0.37
	At second and third trimester (per 1 SD, kg/week)		0.96 (0.61–1.51)	0.85
	Low, *n* (%)	35/92 (14.8)	1.18 (0.78–1.78)	0.42
	Normal, *n* (%)	72/181 (30.4)	Ref	
	High, *n* (%)	130/314 (54.8)	1.01 (0.69–1.48)	0.95

Normal, low and high pre-pregnancy BMI represented the BMI < 18.5 kg/m*^2^*, 18.5–24.0 kg/m*^2^*, and ≥ 24.0 kg/m*^2^*.

In the IoL method 1 subgroup, each 1 kg/m^2^ increase in pre-pregnancy BMI was associated with a 6% increased risk of CDaI, with an aRR (95% CI) 1.06 (1.02–1.11), and *p* = 0.006 (Table 4). Compared with participants with normal pre-pregnancy weight, those who were underweight had a 42% lower risk of CDaI, with an aRR (95% CI) of 0.58 (0.35–0.98), and *p* = 0.041. In the IoL method 2 subgroup, each 1 kg/m^2^ increase in pre-pregnancy BMI was associated with a 5% increased risk of CDaI, with an aRR (95% CI) of 1.05 (1.03–1.08), and *p* < 0.001 (Table 5). Compared to participants with normal pre-pregnancy weight, obese women had a 42% increased risk of CDaI with an aRR (95% CI) of 1.42 (1.02–1.99), and *p* = 0.040. By contrast, underweight participants had a reduced 32% risk of CDaI with an aRR (95% CI) of 0.68 (0.50–0.90), and *p* = 0.012. Each weight increase of 1 SD during the entire pregnancy was associated with a 25% increase in the CDaI risk with an aRR (95% CI) of 1.25 (1.15–1.36), and *p* < 0.001.

**Table 4:** Association analyses for the pregnant women with IoL by method 1 (*n* = 511).

Variables	Cases/total (%)	Adjusted *RR*	*P*
Pre-pregnancy BMI		1.06 (1.02–1.11)	0.006^a^
Underweight (BMI < 18.5 kg/m^2^), *n* (%)	18/61 (29.5)	0.58 (0.35–0.98)	0.041^a^
Normal weight (18.5 kg/m^2^ ≤ BMI < 24 kg/m^2^), *n* (%)	138/335 (41.2)	Ref	
Overweight (24 kg/m^2^ ≤ BMI < 28 kg/m^2^), *n* (%)	56/95 (58.9)	1.21 (0.88–1.66)	0.24^a^
Obese (BMI ≥ 28 kg/m^2^), *n* (%)	12/20 (60.0)	1.37 (0.73–2.56)	0.29^a^
GWG			
At first trimester (per 1 SD, kg)		1.11 (0.96–1.27)	0.16^b^
Inadequate, *n* (%)	55/139 (40.0)	1.17 (0.82–1.68)	0.38^b^
Within guidelines, *n* (%)	79/187 (42.2)	Ref	
Excessive, *n* (%)	90/185 (48.6)	1.32 (0.96–1.79)	0.08^b^
Total pregnancy (per 1 SD, kg)		1.12 (0.98–1.29)	0.08^b^
Inadequate, *n* (%)	63/152 (41.4)	1.14 (0.96–1.34)	0.13^b^
Within guidelines, *n* (%)	86/168 (51.2)	Ref	
Excessive, *n* (%)	75/191 (39.3)	1.09 (0.93–1.26)	0.28^b^
Rate of GWG			
At first trimester (per 1 SD, kg/week)		1.39 (0.87–2.12)	0.16^b^
At second and third trimester (per 1 SD, kg/week)		0.93 (0.55–1.58)	0.80^b^
Low, *n* (%)	69/162 (42.6)	1.05 (0.74–1.51)	0.77^b^
Normal, *n* (%)	61/129 (47.3)	Ref	
High, *n* (%)	94/220 (42.7)	0.98 (0.71–1.37)	0.93^b^

a Adjusted for age, gestational week, ART, epidural labor analgesia, doula-accompanied delivery, GDM and HDP.

b Adjusted for pre-pregnancy BMI, age, gestational week, ART, epidural labor analgesia, doula-accompanied delivery, GDM and HDP; Ref, reference group.

**Table 5:** Association analyses for the pregnant women with IoL method 2 (*n* = 2097).

Variables	Case/total (%)	Adjusted *RR*	*P*
Pre-pregnancy BMI		1.05 (1.03–1.08)	<0.001^a^
Underweight (BMI < 18.5 kg/m^2^), *n* (%)	49/267 (18.3)	0.68 (0.50–0.90)	0.012^a^
Normal weight (18.5 kg/m^2^ ≤ BMI < 24 kg/m^2^), *n* (%)	400/1441 (27.8)	Ref	
Overweight (24 kg/m^2^ ≤ BMI < 28 kg/m^2^), *n* (%)	83/282 (29.4)	1.06 (0.83–1.35)	0.63^a^
Obese (BMI ≥ 28 kg/m^2^), *n* (%)	42/107 (39.2)	1.42 (1.02–1.99)	0.040^a^
GWG			
At first trimester (per 1 SD, kg)		1.05 (0.99–1.10)	0.10^b^
Inadequate, *n* (%)	119/540 (22.0)	0.91 (0.72–1.13)	0.39^b^
Within guidelines, *n* (%)	243/825 (29.4)	Ref	
Excessive, *n* (%)	212/732 (28.9)	1.18 (0.98–1.42)	0.07^b^
Total pregnancy (per 1 SD, kg)		1.25 (1.15–1.36)	<0.001^b^
Inadequate, *n* (%)	153/611 (25.0)	1.16 (0.93–1.43)	0.18^b^
Within guidelines, *n* (%)	200/731 (27.4)	Ref	
Excessive, *n* (%)	221/755 (29.3)	1.18 (0.97–1.42)	0.10^b^
Rate of GWG			
At first trimester (per 1 SD, kg/week)		1.16 (0.97–1.38)	0.10^b^
At second and third trimester (per 1 SD, kg/week)		0.90 (0.66–1.21)	0.45^b^
Low, *n* (%)	164/633 (25.9)	1.21 (0.97–1.51)	0.09^b^
Normal, *n* (%)	160/581 (27.5)	Ref	
High, *n* (%)	250/883 (28.3)	1.06 (0.86–1.29)	0.57^b^

a Adjusted for age, gestational week, ART, epidural labor analgesia, doula-accompanied delivery, GDM and HDP.

b Adjusted for pre-pregnancy BMI, age, gestational week, ART, epidural labor analgesia, doula-accompanied delivery, GDM and HDP; Ref, reference group.

## 4 Discussion

This prospective cohort study examined the associations of pre-pregnancy BMI, GWG, and GWG rate at different pregnancy stages in primiparous individuals with CDaI. We found that increased pre-pregnancy BMIs, high GWGs, and rapid GWGs in the first trimester were associated with 6, 21, and 26% increases in the CDaI risk, respectively. High pre-pregnancy BMI, high GWG, and rapid GWG in the first trimester may be risk factors for CDaI in primiparous women, suggesting that maintaining an appropriate pre-pregnancy BMI and reasonable control of weight increases during pregnancy may decrease the incidence of CDaI in primiparas.

Pregnancy weight has received increasing attention with studies reporting on the associations of pre-pregnancy BMI and GWG with GDM, HDP, offspring development, and other variables (16–18). However, there is a paucity of studies focusing on weight and the CDaI risk, and even fewer among primiparas. The incidence of CDaI in primiparous women is usually higher than in multiparous women (19), and clarifying the mechanisms for this difference is important to reduce the incidence of CDaI. However, studies on the associations of pre-pregnancy BMI and GWG with CDaI have reached different conclusions. In their study, Kwon et al. (20) found that women who were overweight, obese, and extremely obese pre-pregnancy had a significantly increased risk of CDaI, and extremely obese women had the greatest risk. Wolfe et al. (21) found that obesity is an independent risk factor for CDaI, and that the highest risk occurs in women with a BMI greater than 40 kg/m^2^. These findings are consistent with our results. We found that higher pre-pregnancy BMI levels were associated with increased risk of CDaI. Compared with women presenting normal pre-pregnancy weight, obese individuals had a 35% increased risk of CDaI, while underweight individuals had a reduced 31% risk of CDaI. In another prospective cohort study on 6,959 Dutch women (22) the GWGs in the second and third trimesters were not associated with CD. However, in a retrospective cohort study with American Samoan women, Hawley et al. (23) found that GWGs in the second trimester were positively correlated with CD, whereas GWGs in the third trimester showed no correlation. A study by Haile et al. (24) showed that nearly half of the pregnant women in their study had GWGs higher than the recommended guidelines by the Institute of Medicine (IOM), and approximately 13.6% of them underwent CDaI. Women with excessive GWGs had a higher risk of CDaI than those with normal GWGs. However, these studies did not consider the GWG rates, and the study cohorts included more than just primiparas. We found that each 1 SD increase in total GWG was associated with a higher risk of CDaI, no matter the level of pre-pregnancy BMI. In addition, each 1 SD increase in GWG and rate of GWG at the first trimester with CDaI risk were observed in the normal preconception BMI sub-group.

The mechanisms for the association between a high BMI and the risk of CDal have not been fully elucidated. We propose three mechanisms that may be crucial for the association of high BMIs with the CDaI risk: (1) Cephalopelvic disproportion due to macrosomia in obese pregnant women (25). (2) Fat accumulation in the birth canal of obese pregnant women causes tissue thickening and reduction of birth canal space. This in turn would increase the delivery resistance obstructing the head descent and rotation. (3) The high cholesterol and low leptin levels usually associated with high BMIs may have negative effects on uterine contraction strength due to their effects on the oxytocin receptor or calcium channel blockade (26). We also found that the risk of CDaI is decreased in participants with lower pre-pregnancy BMIs compared with those with normal pre-pregnancy BMI.

The delivery method of primiparous women strongly influences their delivery method for subsequent pregnancies. Therefore, studies on pre-pregnancy weight and GWG in primiparous individuals are important. High-fat, high-glucose, and high-calorie foods are now common in daily diets. The habit of increased mobile phone and computer use with insufficient amounts of exercise, fatty, sugary, and high-calorie diets cause difficulties in maintaining pre-pregnancy weights within appropriate ranges. Only approximately 60% of women consider weight control an important part of their pre-pregnancy plans (27). The situation of GWG is also worrying, according to a survey (28), more than half of pregnant women believe that eating without restraint during pregnancy will help the fetus get enough nutrition. Some pregnant women have insufficient knowledge about the importance of exercise during pregnancy, and population educational strategies are needed. GWG can be controlled through lifestyle interventions during pregnancy (29). Regardless of the level of BMI during pregnancy, pregnant women should pay more attention to GWG, especially control the rate of GWG at the first trimester. In 2009, the U.S. IOM published GWG guidelines (30). In addition, the World Health Organization (31) recommends that all pregnant women without contraindications should engage in at least 150 min of moderate-intensity aerobic activity per week during pregnancy as a beneficial intervention to optimize maternal and fetal outcomes. However, excessive GWG is very common among women in all pre-pregnancy BMI categories. Only one-third of pregnant women worldwide fall within the appropriate weight gain ranges (32). A cohort study in Ireland found that only a small fraction of overweight and obese pregnant women met exercise recommendations (33). We studied the associations of pre-pregnancy BMI and GWG rate in different trimesters with the risk of CDaI, to obtain evidence important to promote effective weight management during prenatal or first obstetrics examinations in primiparas to reduce their risk of CDaI.

### 4.1 Strengths and limitations

To the best of our knowledge, this is the first prospective cohort study focusing on the pre-pregnancy BMI, GWGs, and GWG rate during pregnancy in primiparas with CDaI risk, which may have important clinical implications. However, there were some limitations. First, we could not rule out some potential confounding factors in our study. For instance, some pregnant women develop third trimester edema leading to apparent weight gain, but we failed to distinguish these cases from those with weight gains due to other causes. Second, the study participants were all pregnant women from Shanghai, a demographic known to maintain appropriate body weights and living under appropriate economic and medical conditions, leading to potential selection bias. Thus, large multicenter cohort studies are required to determine if our results are representative of those in other regions of China.

## 5 Conclusion

In summary, our results showed for the first time that high pre-pregnancy BMIs and GWGs, as well as rapid GWGs during the first trimester, can significantly increase the CDaI risk in primiparous women. These results demonstrate that achieving reasonable pre-pregnancy BMIs and GWGs may be a safe approach to prevent CDaI in primiparas. Maintenance of normal pre-pregnancy weight and GWG management are crucial for successful IoL in primiparas. Obstetricians should be aware of the potential increased CDaI risk of primiparous women with pre-pregnancy obesity and gestational obesity, particularly in those with excessive first trimester weight gain.

## Data Availability

The raw data supporting the conclusions of this article will be made available by the authors, without undue reservation.

## References

[1] Middleton P Shepherd E Crowther CA. Induction of labour for improving birth outcomes for women at or beyond term. Cochrane Database Syst Rev. (2018) 5:CD004945. doi: 10.1002/14651858.CD004945.pub4, PMID: 29741208 PMC6494436

[2] Martin JA Hamilton BE Osterman MJK Driscoll AK Drake P. Births: final data for 2017. Natl Vital Stat Rep. (2018) 67:1–50. 30707672

[3] Evidence Cafa. New South Wales mothers and babies 2016. Sydney: NSW Ministry of Health (2017).

[4] Tolcher MC Holbert MR Weaver AL McGree ME Olson JE El-Nashar SA. Predicting cesarean delivery after induction of labor among nulliparous women at term. Obstet Gynecol. (2015) 126:1059–68. doi: 10.1097/AOG.0000000000001083, PMID: 26444107 PMC4618703

[5] Asicioglu O Gungorduk K Yildirim G Asicioglu BB Gungorduk OC Ark C. Second-stage vs first-stage caesarean delivery: comparison of maternal and perinatal outcomes. J Obstet Gynaecol. (2014) 34:598–604. doi: 10.3109/01443615.2014.920790, PMID: 24911878

[6] Lumbiganon P Laopaiboon M Gulmezoglu AM Souza JP Taneepanichskul S Ruyan P. Method of delivery and pregnancy outcomes in Asia: the WHO global survey on maternal and perinatal health 2007-08. Lancet. (2010) 375:490–9. doi: 10.1016/S0140-6736(09)61870-5, PMID: 20071021

[7] Garcia-Simon R Montanes A Clemente J Del Pino MD Romero MA Fabre E. Economic implications of labor induction. Int J Gynaecol Obstet. (2016) 133:112–5. doi: 10.1016/j.ijgo.2015.08.022 26868065

[8] D'Souza R Ashraf R Foroutan F. Prediction models for determining the success of labour induction: a systematic review and critical analysis. Best Pract Res Clin Obstet Gynaecol. (2022) 79:42–54. doi: 10.1016/j.bpobgyn.2021.12.005, PMID: 35090827

[9] Danilack VA Hutcheon JA Triche EW Dore DD Muri JH Phipps MG. Development and validation of a risk prediction model for cesarean delivery after labor induction. J Womens Health. (2020) 29:656–69. doi: 10.1089/jwh.2019.7822, PMID: 31657668 PMC8935479

[10] Rossi RM Requarth E Warshak CR Dufendach KR Hall ES DeFranco EA. Risk calculator to predict cesarean delivery among women undergoing induction of labor. Obstet Gynecol. (2020) 135:559–68. doi: 10.1097/AOG.0000000000003696 32028500

[11] Rijal P. Identification of risk factors for cesarean delivery following induction of labour. J Nepal Health Res Counc. (2014) 12:73–7. PMID: 25574996

[12] Society of Perinatal Medicine CMAOS, Society of Obstetrics and Gynecology, Chinese Medical Association. Timing of delivery for pregnancies with comorbidities and complications: expert consensus. Chinese. J Perinat Med. (2020) 23:12. doi: 10.3760/cma.j.cn112141-20200609-00489

[13] Nutrition CSo. Weight monitoring and evaluation during pregnancy period of Chinese women. Chinese Society of Nutrition (2021).

[14] Sterne JA White IR Carlin JB Spratt M Royston P Kenward MG. Multiple imputation for missing data in epidemiological and clinical research: potential and pitfalls. BMJ. (2009) 338:b2393. doi: 10.1136/bmj.b2393, PMID: 19564179 PMC2714692

[15] Obstetrics Subgroup CSoOaG, Chinese Medical Association. Guideline of cervical ripening and labor induction during the third trimester pregnancy. Chin J Obstetr Gynecol. (2014) 49:5. doi: 10.3760/cma.j.issn.0529-567X.2014.12.001 25608986

[16] Teede HJ Bailey C Moran LJ Bahri Khomami M Enticott J Ranasinha S. Association of Antenatal Diet and Physical Activity-Based Interventions with Gestational Weight Gain and pregnancy outcomes: a systematic review and Meta-analysis. JAMA Intern Med. (2022) 182:106–14. doi: 10.1001/jamainternmed.2021.6373, PMID: 34928300 PMC8689430

[17] Robillard PY Dekker G Scioscia M Bonsante F Boukerrou M Iacobelli S. Preeclampsia in 2023: time for preventing early onset-and term preeclampsia: the paramount role of gestational weight gain. J Reprod Immunol. (2023) 158:103968. doi: 10.1016/j.jri.2023.103968 37290173

[18] Chen F Wang P Wang J Liao Z Zong X Chen Y. Analysis and comparison of early childhood nutritional outcomes among offspring of Chinese women under the Chinese 2021 and US 2009 gestational weight gain guidelines. JAMA Netw Open. (2022) 5:e2233250. doi: 10.1001/jamanetworkopen.2022.33250, PMID: 36149650 PMC9508653

[19] Zhu J Xue L Shen H Zhang L Lu D Wang Y. Labor induction in China: a nationwide survey. BMC Pregnancy Childbirth. (2022) 22:463. doi: 10.1186/s12884-022-04760-6, PMID: 35650545 PMC9158355

[20] Kwon HY Kwon JY Park YW Kim YH. The risk of emergency cesarean section after failure of vaginal delivery according to prepregnancy body mass index or gestational weight gain by the 2009 Institute of Medicine guidelines. Obstet Gynecol Sci. (2016) 59:169–77. doi: 10.5468/ogs.2016.59.3.169, PMID: 27200306 PMC4871932

[21] Wolfe KB Rossi RA Warshak CR. The effect of maternal obesity on the rate of failed induction of labor. Am J Obstet Gynecol. (2011) 205:e1–7. doi: 10.1016/j.ajog.2011.03.051 21621187

[22] Gaillard R Durmus B Hofman A Mackenbach JP Steegers EA Jaddoe VW. Risk factors and outcomes of maternal obesity and excessive weight gain during pregnancy. Obesity. (2013) 21:1046–55. doi: 10.1002/oby.20088 23784909

[23] Hawley NL Johnson W Hart CN Triche EW Ah Ching J Muasau-Howard B. Gestational weight gain among American Samoan women and its impact on delivery and infant outcomes. BMC Pregnancy Childbirth. (2015) 15:10. doi: 10.1186/s12884-015-0451-1, PMID: 25643752 PMC4324802

[24] Haile ZT Chavan B Teweldeberhan AK Chertok IRA Francescon J. Gestational weight gain and unplanned or emergency cesarean delivery in the United States. Women Birth. (2019) 32:263–9. doi: 10.1016/j.wombi.2018.07.011 30093348

[25] Walsh J Foley M O'Herlihy C. Dystocia correlates with body mass index in both spontaneous and induced nulliparous labors. J Matern Fetal Neonatal Med. (2011) 24:817–21. doi: 10.3109/14767058.2010.531313, PMID: 21158492

[26] Hajagos-Toth J Ducza E Samavati R Vari SG Gaspar R. Obesity in pregnancy: a novel concept on the roles of adipokines in uterine contractility. Croat Med J. (2017) 58:96–104. doi: 10.3325/cmj.2017.58.96, PMID: 28409493 PMC5410735

[27] Bahri Khomami M Walker R Kilpatrick M de Jersey S Skouteris H Moran LJ. The role of midwives and obstetrical nurses in the promotion of healthy lifestyle during pregnancy. Ther Adv Reprod Health. (2021) 15:26334941211031866. doi: 10.1177/26334941211031866 34396131 PMC8361518

[28] Sugiyama T Nagao K Metoki H Nishigori H Saito M Tokunaga H. Pregnancy outcomes of gestational diabetes mellitus according to pre-gestational BMI in a retrospective multi-institutional study in Japan. Endocr J. (2014) 61:373–80. doi: 10.1507/endocrj.ej13-0541, PMID: 24476982

[29] Group IWMiPi-WC. Effect of diet and physical activity based interventions in pregnancy on gestational weight gain and pregnancy outcomes: meta-analysis of individual participant data from randomised trials. BMJ. (2017) 358:j3991. doi: 10.1136/bmj.j3991, PMID: 28835414 PMC5566764

[30] Rasmussen KM Yaktine AL eds. (US) NAP In: Weight gain during pregnancy: reexamining the guidelines: The National Academies Collection: Reports funded by National Institutes of Health. Washington, DC: (2009) 20669500

[31] Bull FC Al-Ansari SS Biddle S Borodulin K Buman MP Cardon G. World Health Organization 2020 guidelines on physical activity and sedentary behaviour. Br J Sports Med. (2020) 54:1451–62. doi: 10.1136/bjsports-2020-102955, PMID: 33239350 PMC7719906

[32] Gilmore LA Klempel-Donchenko M Redman LM. Pregnancy as a window to future health: excessive gestational weight gain and obesity. Semin Perinatol. (2015) 39:296–303. doi: 10.1053/j.semperi.2015.05.009, PMID: 26096078 PMC4516569

[33] O'Keeffe LM Dahly DL Murphy M Greene RA Harrington JM Corcoran P. Positive lifestyle changes around the time of pregnancy: a cross-sectional study. BMJ Open. (2016) 6:e010233. doi: 10.1136/bmjopen-2015-010233, PMID: 27154477 PMC4861121

