# Supplementary material for: Associations of primiparous pre-pregnancy body mass index and gestational weight gain with cesarean delivery after induction: a prospective cohort study
Source: Front Med (Lausanne). 2024 Aug 30;11:1453620. doi: 10.3389/fmed.2024.1453620 (PMC11392890; doi:10.3389/fmed.2024.1453620)
Supplement: Supplementary file 1 [file Data_Sheet_1.docx]

Supplementary Material

**Table S1.** The criteria of pre-pregnancy BMI, weight gain ranges during pregnancy and recommended weekly weight gains in the second and third trimesters.

| Classification of pre-pregnancy BMI | Range of total GWG (kg) | Range of GWG at first trimester (kg) | Mean and range of GWG rate at second and third trimester (kg/week) |
| --- | --- | --- | --- |
| Underweight (BMI < 18.5 kg/m^2^) | 11.0–16.0 | 0–2.0 | 0.46 (0.38–0.56) |
| Normal weight (18.5 kg/m^2^ ≤ BMI < 24.0 kg/m^2^) | 8.0–14.0 | 0–2.0 | 0.37 (0.26–0.48) |
| Overweight (24.0 kg/m^2^ ≤ BMI < 28.0 kg/m^2^) | 7.0–11.0 | 0–2.0 | 0.30 (0.22–0.37) |
| Obese (BMI ≥ 28.0 kg/m^2^) | 5.0–9.0 | 0–2.0 | 0.22 (0.15–0.30) |

BMI, body mass index; GWG, gestational weight gain.

**Table S2.** The definitions of different induction of labor methods in this study.

| IoL method | Definitions |
| --- | --- |
| IoL method 1 | For women with a cervical Bishop score ≥ 6 points, artificial rupture of membranes was conducted, and then an intravenous infusion of oxytocin was administered after 2 hours of irregular uterine contraction. |
| IoL method 2 | For women with a cervical Bishop score between 4 and 5 points, a Foley bulb was placed for cervical ripening. After 12 hours of irregular uterine contractions, we conducted manual rupture of membranes immediately after removing the bulb, and then an intravenous infusion of oxytocin was administered after another 2 hours of irregular uterine contractions. |
| IoL method 3 | For women with cervical Bishop score ≤3 points, a dinoprostone suppository was placed in the posterior vaginal fornix; and the suppository was removed once the cervical Bishop score reached ≥6 points or the uterine contractions were frequent. Artificial rupture of membranes or intravenous infusion of oxytocin were procedures performed according to the specific circumstances. |

IoL, induction of labor

# Supplementary Data

Supplementary Material should be uploaded separately on submission. Please include any supplementary data, figures and/or tables.

Supplementary material is not typeset so please ensure that all information is clearly presented, the appropriate caption is included in the file and not in the manuscript, and that the style conforms to the rest of the article.

# Supplementary Figures and Tables

For more information on Supplementary Material and for details on the different file types accepted, please see [here](https://www.frontiersin.org/guidelines/author-guidelines#supplementary-material).

## Supplementary Figures

**
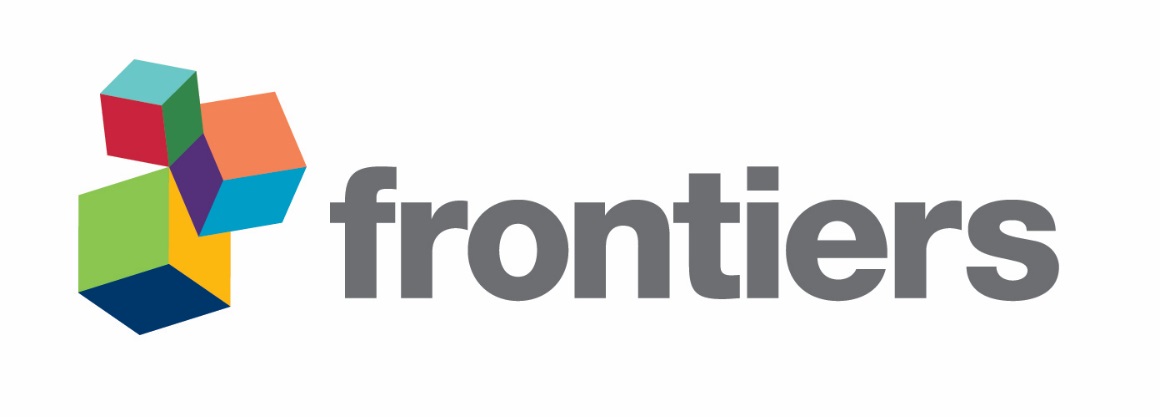
**

**Supplementary Figure 1.** The figure legends are required to have the same font as the main text, 12 point normal Times New Roman, single spaced. Please use a single paragraph for each legend and prepare the figures keeping in mind the PDF layout.
